# Supplementary figures and images for: Effects of natural treatments on the varroa mite infestation levels and overall health of honey bee (Apis mellifera) colonies
Source: PLoS One. 2024 May 7;19(5):e0302846. doi: 10.1371/journal.pone.0302846 (PMC11075888; doi:10.1371/journal.pone.0302846)

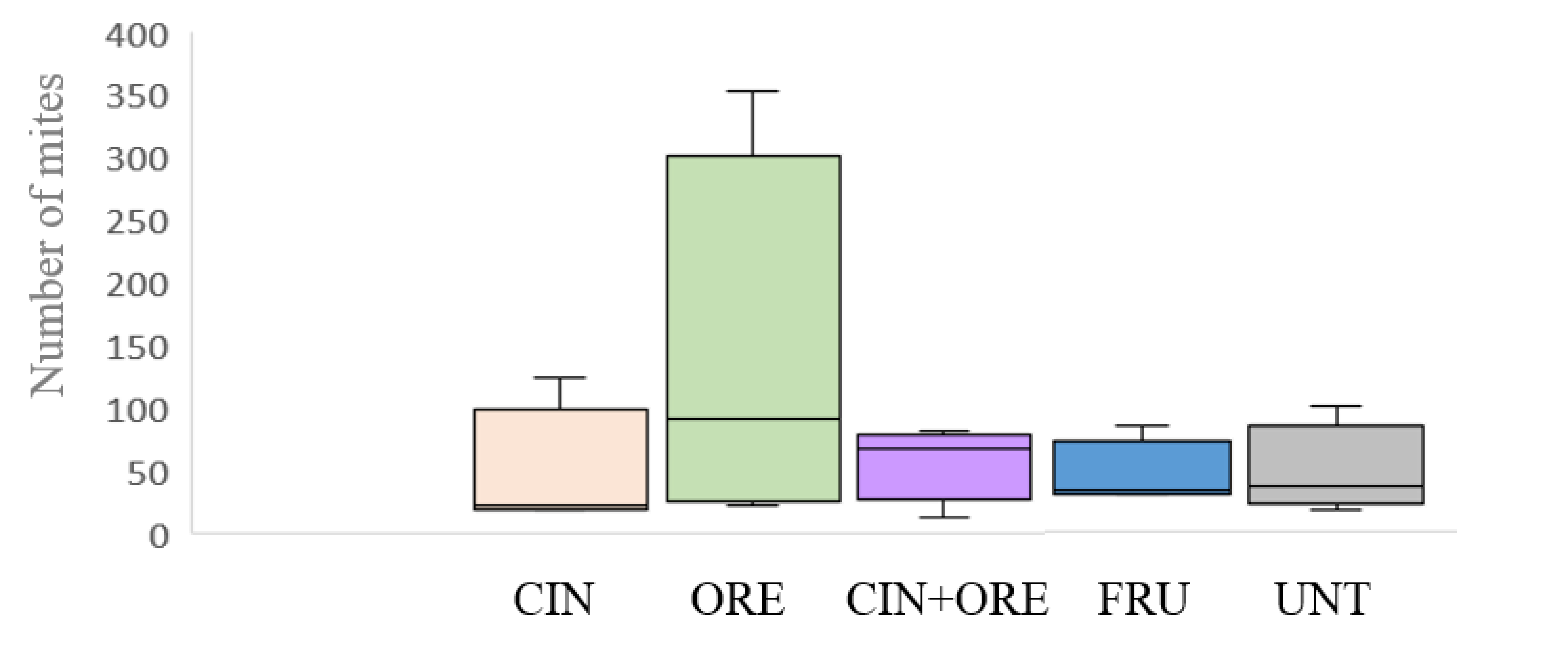

Supplement: S1 Fig — The Fig shows the normalized median mite quantities and interquartile ranges in both control and treatment groups. Number of mites trapped on sticky boards 24 hours after the treatment with Api Bioxal (T86). The groups are: CIN, cinnamon EO-supplemented syrup; ORE, oregano EO-supplemented syrup; CIN+ORE, cinnamon and oregano EOs-supplemented syrup; FRU, juice cocktail-supplemented syrup; UNT, unsupplemented syrup. Between-group analysis was performed by the Kruskal-Wallis test followed by the Dunn’s multiple comparisons test. Data including minimum score, 1st quartile, median, 3rd quartile and maximum score are shown for each group. (TIF) [file pone.0302846.s001.tif]
