# Supplementary material for: Effects of natural treatments on the varroa mite infestation levels and overall health of honey bee (Apis mellifera) colonies
Source: PLoS One. 2024 May 7;19(5):e0302846. doi: 10.1371/journal.pone.0302846 (PMC11075888; doi:10.1371/journal.pone.0302846)
Supplement: Attachment — Submitted filename: Second Response to Reviewers.docx [file pone.0302846.s004.docx]

**Reply to Reviewer 2**

**Q1**: Line 115: The experimental design had a treatment that has five levels, the control group is one more level of the treatment. This may seem like a minor point and does not affect the coherence of the study, but it does address the use of terminology specific to the experimental design.

**R:** We have taken note of the clarification and have amended the text accordingly.

**Q2:** The authors state that they do not know the frequency distribution of the response variables. This is obvious before carrying out the study, then with the data obtained they evaluated whether they met the assumption of normality. Not being proven, they decided, correctly, to use non-parametric methods. I believe that the statistical approach used by the authors is not entirely correct, since, with the frequency distribution they can evaluate which distribution they can fit, choose the appropriate link function, and use a Generalized Linear Model, which is a method much more appropriate.

On the other hand, it is possible to incorporate into generalized linear models the random effect of having measured the same hive on several occasions throughout the trial. I suggest that the authors, evaluate their data using a Generalized Linear Model of repeated measures, using the distribution that best fits the observed data, and select the appropriate link function. I consider that the statistical method used by the authors is not wrong, it is just not the most appropriate.

**R:** We agree that GLM would be the most appropriate method to analyse this type of data, provided that the distribution of the data is known or can be determined with confidence. In our experiment, the number of hives per group was rather small (n=4), and we did not feel confident to base the determination of the data distribution and, consequently, of the link function, whose choice affects the reliability of the GLM results, on such a limited number of hives. For this reason, we preferred to use the non-parametric method, which is reliable regardless of the exact identification of the data distribution.

**Q3**. I understand the authors' concern about the size of each treatment level, undoubtedly one of the most relevant limitations of the study. However, once the limitation was recognized, what I requested in the previous review was that they support the sample size used. I understand the authors about they did not have an expected effect size before the trial and they used similar approaches from other works. This is not wrong in itself, but it does not fully clarify the point. When you design an experiment, you establish the sample size and if you do not have an idea of the expected effect, you can make a priori hypotheses. However, if the sample size was decided based on previous studies, what I requested is that you estimate the power of the design used, which can provide evidence of the limitation. The power calculation will tell you the probability that they made a Type II error.

**R:** The sample size per group used in our study (n=4) would have allowed us to detect an effect of very large magnitude (Cohen's d = 3.2) with multiple comparisons performed by the Mann-Whitney U test (min ARE distribution), with one-tailed alpha = 0.0125 (i.e., experimentwise alpha = 0.05 using Bonferroni's correction for 4 multiple comparisons) and power = 0.80. Under the same conditions of alpha, tails and statistical test, the power to detect an effect of medium to large size (Cohen's d = 1) would have been extremely low (1-beta = 0.11). In any case, the differences in mite infestation levels between the treatment and control groups were very small and mostly in the opposite direction to what we expected, suggesting a lack of efficacy of our treatments. This finding suggests that the lack of significance in the differences is due to a real lack of effect rather than a problem of low power. To give you a complete picture, we would have needed 24 hives per group (120 hives in total) to detect a Cohen's d effect size of 1.0, using the same statistical parameters (one-tailed alpha = 0.0125 and power = 0.80), making it impossible to carry out the desired experiment.

**Reply to Reviewer 3**

**Q**: The authors responded to the comments and revised much of what was requested. I still have some comments, which had not been fully resolved previously.

- Line 165: the percentages of the different fruits that make up the juice have been included, but in my opinion the analytical composition (e.g. vitamins, sugars, minerals, fibres...) is still missing in order to know what was actually fed to the bees.

**R1:** We added the nutritional information of Cuore di Frutta, as it appears on the label of the juice. Please, see lines 166-166. There is no nutritional information on the blueberry juice.

- Line 174: the concentration of oxalic acid administered with the treatment is still missing. It is indicated on the product label.

**R2**: We included the oxalic acid concentration.

- Line 215: the authors stated that they chose the pathogens most prevalent in the apiary. Do they refer to previous pathological analyses? Do they refer to literature data?

**R3**: The literature was cited and we replaced the term 'the most prevalent' with 'common'.

- Line 348: I still do not understand the 'background mortality'. It is mentioned here for the first time, but how is it measured? Where is the explanation of its measurement and statistical analysis in the materials and methods?

**R4:** The number of dead bees inside each hive was recorded 24 hours after the first treatment. No significant differences were found compared to the control group, which experienced background mortality. However, measurements were not repeated after the second and third treatments. We did not mention this in the Materials and Methods section as it was outside the scope of our research. As we did not thoroughly investigate the toxicity of our treatments towards bees, we removed any references to the safety of the tested treatments from the abstract, methods, and discussion.

.
- Line 489-490: why does the reader have to get to the bottom of the article to know the reason for the choice of essential oils?

**R5:** We agree with the reviewer and relocated the sentence in the Introduction section. Please, see lines 94-96.

- I am still unsure about the method of estimating the amount of honey produced. There was no need to extract the honey, it would have been sufficient to weigh the empty supers and reweigh them again once they were filled with honey.

**R6:** We agree with the reviewer that our estimation of honey production was inaccurate. Unfortunately, we are unable to rectify this. However, if the reviewer believes that our method is fundamentally flawed, we are willing to remove any references to honey production from the text.

- Paray et al. (doi.org/10.1016/j.sjbs.2020.11.053) reviewed honey bee nutrition and pollen sobstitutes. There is also a reference to a work where different fruit juices were fed to bees. Perhaps this could be useful for introduction and/or discussion.

**R7:** We greatly appreciate the reviewer's suggestion. In our introduction, we referenced the study conducted by Pande et al. (2015), which examined the use of four different fruit juices as supplements for bee feeding. Please, see lines 85-87 and 92-93.
